# Evaluation of a Microhaplotype Marker Panel for Post-Transplant Chimerism Analysis

**DOI:** 10.1159/tmh/aevag001

**Published:** 2026-08-03

**Authors:** Christian Lang, Gabi Rink, Lars Lodder, Julia Wentzell, Birgit Omengo, Peter Bugert

**Affiliations:** a Institute of Transfusion Medicine and Immunology, Medical Faculty Mannheim, Heidelberg University, German Red Cross Blood Service Baden-Württemberg - Hessen, Mannheim, Germany; b inno-train Diagnostik GmbH, Kronberg, Germany

**Keywords:** Microhaplotypes, Biomarker, Liquid biopsy, Next-generation sequencing, Genetic marker panel

## Abstract

**Introduction:**

Microhaplotypes (MHs) are highly informative genetic markers suitable for forensic and clinical applications, including post-transplant monitoring in liquid biopsy. The aim of this study was to establish and evaluate a highly informative MH marker panel suitable for both chimerism analysis after allogeneic stem cell transplantation and for monitoring organ transplants using cell-free DNA (cfDNA). Two sequencing platforms (Illumina MiSeq and Oxford Nanopore Technologies [ONT]) were compared with regard to their suitability for reliable MH genotyping.

**Methods:**

Based on 98 candidates selected from the literature, a panel of 74 MH markers was developed and sequenced in 960 anonymized samples of healthy blood donors. Amplification was performed from genomic DNA in two multiplex PCRs generating short amplicons (95–150 bp). Library preparation and sequencing were carried out on the Illumina MiSeq platform (2 × 150 bp) and the ONT MinION (R10.4.1 flow cells). Bioinformatic analysis included quality filtering, read mapping (Bowtie2 and Minimap2), haplotype determination, and calculation of population genetic parameters such as polymorphism information content (PIC), power of discrimination (PD), expected heterozygosity (He), effective number of alleles (A_e_), and Hardy-Weinberg equilibrium.

**Results:**

Evaluation of the sequencing results from 960 samples led to the exclusion of 20 MH markers due to low coverage or nonspecific amplifications. The remaining 54 MH loci were located on 22 chromosomes and comprised a total of 202 single nucleotide variations (SNVs). A balanced allele coverage ratio (>0.6) was observed for 43 loci. In total, 358 different alleles were identified, with most markers exhibiting between four and seven alleles. The PD exceeded 0.6 in 52 loci (mean 0.87), and most markers demonstrated high PIC and He values, indicating substantial genetic variability. Platform comparison revealed that ONT sequencing generated higher absolute read numbers, whereas Illumina sequencing achieved higher relative mapping efficiency and base accuracy. The proportion of uniquely mapped reads was comparable between the platforms. Additionally, previously undescribed SNVs were identified in 23 loci, potentially, further increasing haplotype diversity.

**Conclusion:**

The marker panel with 54 MHs demonstrated high genetic diversity and discriminatory power in the studied population. With the small amplicon size the MH marker panel could also be suitable for cfDNA. Further investigation with regard to the analysis of mixed DNA samples will support the usefulness of MHs as biomarkers for chimerism analysis in post-transplant monitoring.

## Introduction

Microhaplotypes (MHs) represent highly informative genetic markers that have gained increasing attention for forensic and population genetic applications in recent years [1, 2]. MHs are defined as clusters of two or more adjacent single nucleotide variations (SNVs) within a short DNA region of usually less than 200 bp. By combining multiple SNVs within an MH marker, the polymorphic information content (PIC) is significantly increased. Together with the low mutation rate and short sequence lengths, MHs are biomarkers with a high power of discrimination (PD) enabling a reliable resolution of individual profiles and population structures [3, 4]. Unlike short tandem repeat polymorphisms, which rely on length polymorphisms and are prone to mutations and stutter artifacts, MHs are sequence-based and, thus, exhibit greater robustness and analytical precision. These properties make MHs particularly suitable for the analysis of challenging clinical and forensic samples, including mixtures, degraded DNA, and low-template samples. Furthermore, MHs have demonstrated significant potential in biogeographic ancestry inference and kinship testing [5–7]. The implementation of next-generation sequencing (NGS) and third-generation sequencing technologies has greatly facilitated the identification and characterization of MHs. Such high-throughput sequencing enables the simultaneous analysis of multiple loci, providing detailed genetic insights [8, 9].

The characteristics of polymorphic genetic markers render MHs particularly suitable for applications requiring sensitive and specific detection of mixed genetic profiles [10, 11]. In allogeneic hematopoietic stem cell transplantation, genomic DNA (gDNA) obtained from a blood sample of the patient is used for post-transplant monitoring by chimerism analysis. In organ transplantation, chimerism analysis of cell-free DNA (cfDNA) in the plasma is attracting attention for early detection of allograft rejection [12]. Owing to their multi-allelic nature and low mutation rates, MHs enable reliable discrimination between two individuals even at low levels of admixture [13, 14]. MHs as biomarkers in liquid biopsy have the potential to meaningfully complement existing molecular genetic approaches in post-transplant monitoring.

Here, we established and evaluated a panel of 54 highly informative MH markers. The allele frequencies were determined in 960 anonymized blood donor samples and were used for the calculation of population genetic measures. Sequencing was performed on Illumina (MiSeq) and Oxford Nanopore Technologies (ONT; MinION) platforms in order to compare the performance of both technologies regarding inter-locus balance and allele coverage ratio (ACR).

## Methods

### Blood Samples and DNA Isolation

Anonymized blood samples of 960 blood donors were collected at the German Red Cross Blood Service Baden-Württemberg-Hessen, Mannheim, Germany. gDNA was isolated from EDTA blood samples using the QIAamp DNA Blood Mini Kit according to the manufacturer’s instructions. DNA concentration was determined by fluorometry (Quantus™ Fluorometer with QuantiFluor™ ONE dsDNA System; Promega GmbH, Walldorf, Germany).

### MH Selection

Initial selection from the literature included 98 MH candidates [9, 10, 15–19]. The MH loci were selected based on the following criteria: (1) the presence of at least three SNVs in a locus; (2) a maximum distance of 80 bp between the outer SNVs; (3) a large distance between loci on a chromosome; (4) reported high allelic diversity. After primer design for small amplicons (≤150 bp), singleplex tests, and optimization, the final panel included 74 MH markers.

### Primer Design and Multiplex Amplification

Primers were designed to target the genomic sequence (human genome GRCh37, hg19) of the MHs in our panel and validated using Geneious Prime (Geneious 2023.2.1, Biomatters, Ltd., New York, USA). Primer design was achieved for 80 of the 98 initially selected MHs. To ensure specific amplification, the primers were checked using Primer-BLAST. Primer melting temperatures (Tm) ranged from 59 to 65°C; GC content was maintained at 40–60%. Primer length varied between 18 and 24 nucleotides, producing amplicons ranging from 95 to 150 bp (online suppl. Table 1; for all online suppl. material, see https://doi.org/10.1159/tmh/aevag001), suitable also for amplification from cfDNA. The synthesized primers were first tested in singleplex PCR, and primers with poor performance were discarded from the panel. Singleplex amplification was insufficient or nonspecific for 6 MHs which were excluded from further investigation.

Primers for the amplification of the remaining 74 MH loci were successfully multiplexed in two reactions. PCR was performed in a total volume of 30 µL containing 2 µL gDNA (50 ng/µL), 10 µL primer mix (300 nm per Primer), 15 µL Q5^®^ Hot Start High-Fidelity 2X Master Mix (New England Biolabs, Ipswich, MA, USA), and 3 µL nuclease-free water. The PCR program was (1) initial denaturation for 30 s at 98°C; (2) 30 cycles of 7 s at 98°C, 20 s at 63°C, 20 s at 72°C; and (3) final extension for 2 min at 72°C. Multiplex amplification and sequencing of 74 MHs was performed for 960 DNA samples.

### Library Preparation and Sequencing

The PCR products were purified with NucleoMag^®^ B-Beads (Macherey-Nagel, Düren, Germany). A bead-to-sample ratio of 1.8:1 was used. After clean-up, libraries were prepared using NEBNext^®^ NEBNext UltraExpress library prep kits for Illumina (New England Biolabs, Ipswich, MA, USA). We used NEBNext^®^ Multiplex Oligos for Illumina (New England Biolabs, Ipswich, MA, USA) for indexing samples. The final library was equimolarly pooled and sequenced using the Illumina MiSeq 2 × 150 bp sequencing platform (MiSeq Reagent Kits v2, Illumina, San Diego, CA).

Native Barcoding Kit SQK-NBD114.96 (ONT; Oxford, UK) was used to prepare the sequencing library for Oxford nanopore sequencing. For end-preparation ligation, the DNA was adjusted to a total amount of 400 fmol (instead of being diluted to 200 fmol) to ensure a higher input quantity for subsequent steps. Sequencing was performed on an ONT MinION Mk1B device using either a Flongle Flow Cell (R10.4.1) or a MinION Flow Cell (R10.4.1). Flow cells were prepared according to the manufacturer’s instructions to stabilize pore activity and maximize loading efficiency. Loading was carried out with a purified sample volume adjusted to the previously determined DNA concentration. For very short libraries (<1,000 bp), a loading concentration of 35–100 fmol DNA was recommended to achieve optimal sequencing performance.

### Bioinformatic Data Analysis

A fully automated data analysis pipeline was developed to ensure standardized, reproducible, and scalable processing of the large dataset from 960 sequenced samples. Following initial data preprocessing and quality control, raw data were converted into a uniform format and directly exported into an Excel-based analytical environment, which served as an interface between the automated data processing workflow and downstream statistical analysis. To evaluate sequencing performance, reads from both platforms were aligned to the MH reference sequences (hg19) using Bowtie2 (version 2.5.4) with a highly sensitive local alignment strategy. Illumina paired-end reads were aligned in paired mode, whereas ONT reads were aligned in single-end mode. For comparative analysis, only primary alignments were retained by excluding unmapped, secondary, supplementary, and QC-failed reads (-F 0 × 904), followed by sorting and indexing. No additional mapping-quality (MAPQ) or base-quality (BaseQ) thresholds were applied at this stage in order to avoid introducing platform-dependent bias. Alignment statistics, including total and mapped reads, were derived from the processed alignment files and alignment summary reports. For each Illumina paired-end sample, 100,000 reads were sampled from both the R1 and R2 FASTQ files, and for each ONT sample, 100,000 reads were sampled per barcode FASTQ file. Mean read length and mean Phred quality scores were calculated from these subsamples.

The SNVs of each MH marker were analyzed using an in-house bioinformatics pipeline. Input data included MH loci (BED), reference sequences (FASTA), and mapped sequencing reads (SAM). Allele frequency analysis and genotype inference were performed using Illumina sequencing data only. Following primary alignment filtering, reads were further restricted to those overlapping MH SNV positions as defined in the BED file. Off-target mappings and reads not fully covering all MH-defining SNV positions were excluded. Stringent quality thresholds were applied, with minimum values of MAPQ ≥30 and BaseQ ≥30. For each retained locus, SNV allele combinations and their coverage were determined. Zygosity was inferred from the relative frequencies of the two most abundant allele combinations: loci were classified as heterozygous if the major allele combination comprised ≤70% of reads and the minor allele combination ≥15%; otherwise, loci were classified as homozygous. Allele combinations were annotated as major or minor variants, and summary statistics were exported for downstream analysis.

Population genetic parameters were calculated from the resulting allele frequencies. FASTQ files were additionally used in the Geneious Prime 2023.2.1 software (Biomatters, Ltd., New York, USA) to determine the haplotypes and the overall panel performance. Analyses of haplotype frequencies and population parameters included expected heterozygosity (H_e_), Hardy-Weinberg equilibrium (HWE), PD, polymorphism information content (PIC), allele frequency (pi), A_e_, and ACR. The allelic coverage ratio (ACR) is defined as the ratio of the lower sequencing coverage to the higher sequencing coverage of the two alleles at a heterozygous locus within a single sample and is used to assess allelic balance in genetic markers detected by high-throughput sequencing. The PD is the probability that two randomly selected, unrelated individuals possess different genotypic profiles at a given genetic locus and, therefore, represents a measure of the practical discriminating power of a genetic marker. Analysis was performed using an in-house Python script. The values were calculated according to the following formula: *PIC = 1 −*$\Sigma_{i=1}^{k}{p_{i}}^{2}$*−*$\Sigma_{i=1}^{n-1}$$\Sigma_{j=i+1}^{n}\;{{2p}_{i}}^{2}{p_{j}}^{2}$*; A*_*e*_*= 1/*$\Sigma_{i=1}^{k}{p_{i}}^{2}$*; H*_*e*_*= 1 −*$\Sigma_{i=1}^{n}{p_{i}}^{2}$*; RMP = 1 − PD, PD = 1 − RMP,* where p_i_ represents the frequency of the *i*th allele at a given locus [3, 20].

## Results

### Performance Validation of the MH Panel

Sequencing of 74 MHs in 960 DNA samples revealed a low coverage (<50 reads) for 16 loci, which were excluded from further investigation. MH-9, MH-23, MH-41, and MH14CP004 were later removed from the panel due to nonspecific amplification. The remaining 54 MH loci were located on 22 chromosomes (further information given in online suppl. Table 2). The number of SNVs per MH ranged from 3 to 6, and the distance between the outer SNVs varied from 11 bp (MH-20) to 77 bp (15qC), with an average distance of 46 bp (shown in Table 1). Overall, the dataset contained 202 SNVs. The amplicon size was 95–150 bp (mean ± SD 122.3 ± 14.8 bp). The ACR was determined for all heterozygous loci and was calculated as the ratio of the read counts of the two alleles (shown in Fig. 1 and values listed in online suppl. Table 3). For 43 of 54 MHs, the ACR was >0.6 (range 0.61–0.83), indicating a balanced allele coverage. The ACR of 11 MHs was <0.6 (range 0.38–0.59). Further information about the MH loci and the SNVs is summarized in the online supplementary material files.

**Table 1.** Features of the 54 MH loci and number of alleles identified in 960 individuals

MH marker	SNVs, *n*	Chromosome position of outer SNVs (hg19)	Distance between outer SNVs (bp)	Amplicon size (bp)	Alleles, *n*
1qD	3	1:235766960–235766976	16	130	5
3pA	4	3:5534417–5534485	68	150	9
3qB	5	3:42924625–42924691	66	138	4
4pA	4	4:8122461–8122537	76	141	8
4qB	3	4:100042236–100042262	26	123	4
4qC	4	4:155794591–155794641	50	110	7
5qC	3	5:132916359–132916408	49	107	7
6qA	3	6:77943041–77943057	16	112	4
6qB	4	6:96372323–96372356	33	102	7
6qC	3	6:120130340–120130387	47	111	5
7qA	3	7:68052881–68052934	53	126	6
8pB	5	8:40664194–40664243	49	120	9
8qA	3	8:62944955–62945023	68	140	5
8qB	3	8:87160379–87160417	38	105	6
9qB	4	9:104903827–104903863	36	139	6
9qC	5	9:136768837–136768914	54	134	9
10pA	3	10:10412217–10412243	26	140	4
10qB	3	10:83182147–83182163	16	108	5
11pB	4	11:34437364–34437384	20	106	7
11qC	3	11:129277278–129277314	36	104	6
12pB	3	12:30147546–30147571	25	95	4
13qA	4	13:33553580–33553649	69	138	12
14qC	3	14:76633957–76634011	54	118	4
14qD	3	14:98228822–98228846	24	129	5
15qB	3	15:61765468–61765526	58	127	7
15qC	3	15:80584597–80584674	77	121	7
15qD	4	15:98255928–98255978	50	123	4
18pA	5	18:5280017–5280093	76	127	5
18qC	4	18:63842524–63842563	39	136	5
19pA	3	19:561779–561816	37	135	5
19pB	5	19:16151675–16151740	65	127	7
20pA	4	20:8810049–8810123	74	149	5
20qA	3	20:38030332–38030353	21	107	5
22qB	5	22:49060976–49061028	52	112	9
MH01CP007	3	1:12849033–12849049	16	107	3
MH01CP012	3	1:61465284–61465314	30	108	5
MH04CP002	3	4:24306576–24306595	19	100	5
MH05CP004	3	5:9565130–9565156	26	120	4
MH05CP006	3	5:2136570–2136606	36	111	6
MH06CP007	3	6:4383313–4383355	42	118	7
MH06CP013	3	6:14576839–14576915	76	131	6
MH07CP004	5	7:16828694–16828739	45	104	13
MH11CP005	3	11:23357289–23357320	31	114	5
MH14CP012	4	14:33539713–33539775	62	130	6
MH15CP004	3	15:43802024–43802071	47	130	5
MH17CP006	3	17:15289086–15289135	49	139	7
MH-4	3	1:218265683–218265718	36	109	4
MH-5	4	1:228465346–228465403	58	139	7
MH-20	5	6:42614554–42614564	11	139	6
MH-22	6	6:152930711–152930771	61	122	8
MH-25	3	9:565917–565986	70	135	6
MH-27	6	10:47631395–47631462	68	132	24
MH-31	6	12:64140424–64140491	68	121	15
MH-43	4	17:31320658–31320713	56	117	9

**Fig. 1.**
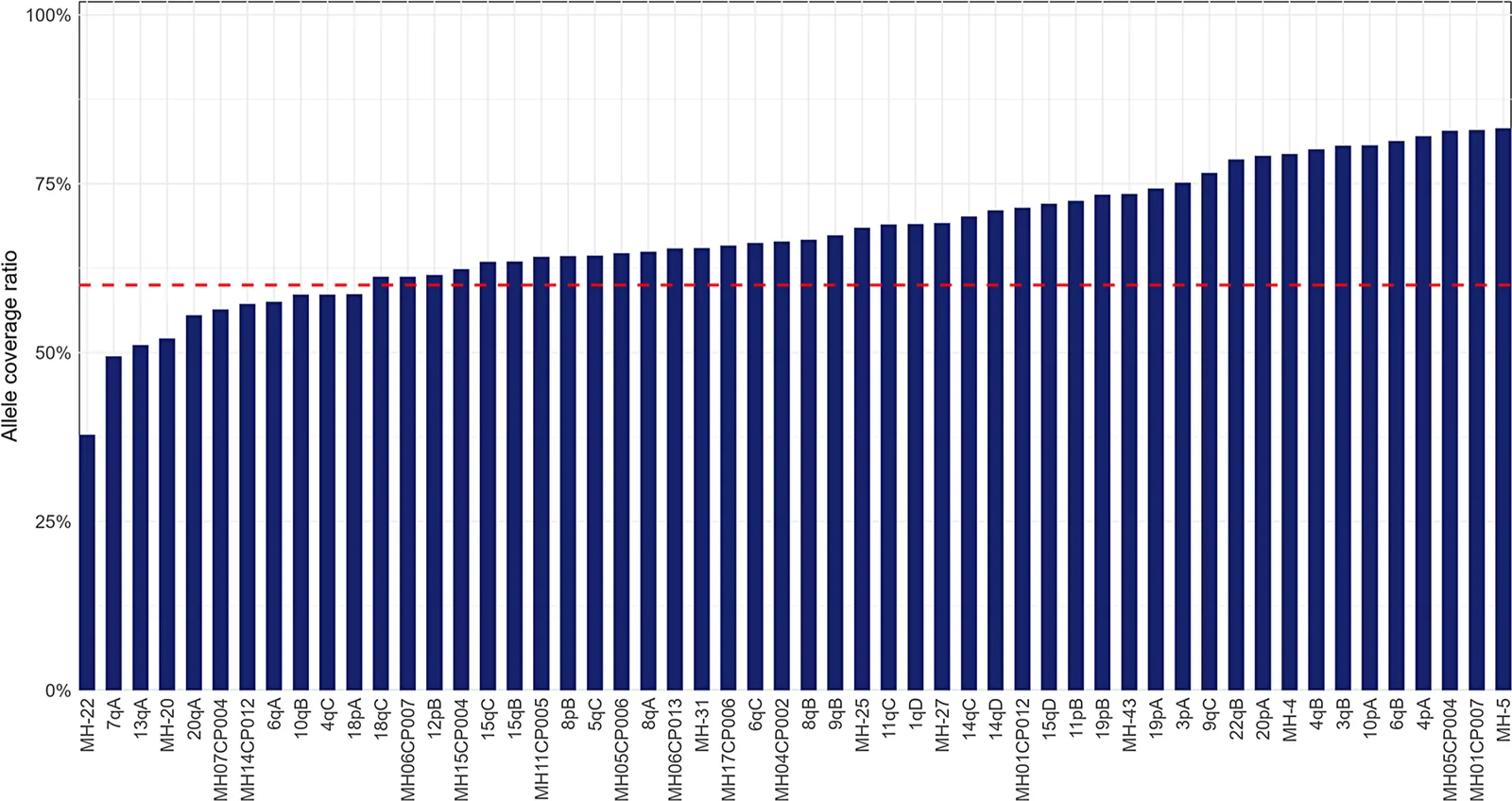
The allele coverage ratio (ACR) in the 960 analyzed samples. Mean ACR (%) per sample, sorted left to right in ascending order. The ACR value of 0.6 is indicated by the dotted line.

For a comparative analysis of sequencing performance, 24 samples were sequenced using both Illumina and ONT platforms. The total number of reads (shown in Fig. 2a) exhibited a considerably higher spread for ONT, with a median of approximately 93,727 reads, an interquartile range (IQR) of about 28,888 reads, and whiskers spanning from 46,987 to 137,755 reads. In comparison, Illumina showed a median of 39,054 reads with a larger IQR of 34,311 and whiskers ranging from 13,373 to 72,897 reads. Mapped reads (shown in Fig. 2b) followed a similar trend: ONT reached a median of 73,609 reads with an IQR of 19,662 and whiskers between 39,685 and 110,536 reads. Illumina’s median was 34,901 with an IQR of 30,701 and whiskers from 11,866 to 64,412 reads. The percentage of mapped reads (shown in Fig. 2c) was higher for Illumina, with a median of 88.94% and a comparatively narrow IQR of 1.98%, compared to ONT’s median of 79.86% and a wider IQR of 6.16%. Whiskers ranged from 86.76% to 92.17% for Illumina and 68.68%–85.77% for ONT, indicating greater variability in ONT data. For uniquely mapped reads (shown in Fig. 2d), medians were nearly identical between platforms: 76.48% for Illumina and 76.68% for ONT; however, ONT exhibited a substantially larger IQR of 6.29% compared to 3.38% for Illumina. Average read length (shown in Fig. 2e) was very consistent for Illumina at a constant 151 bp, whereas ONT produced significantly longer reads with a median of approximately 296 bp, an interquartile range of ∼15 bp, and whiskers spanning 276 to 307 bp. Finally, the mean Phred quality score (Fig. 2f), an indicator of sequencing accuracy, was notably higher for Illumina, with a median of 36.44 compared to 34.34 for ONT. The IQR for ONT (0.812) was larger than for Illumina (0.412), reflecting greater variability in read quality on the Nanopore platform.

**Fig. 2.**
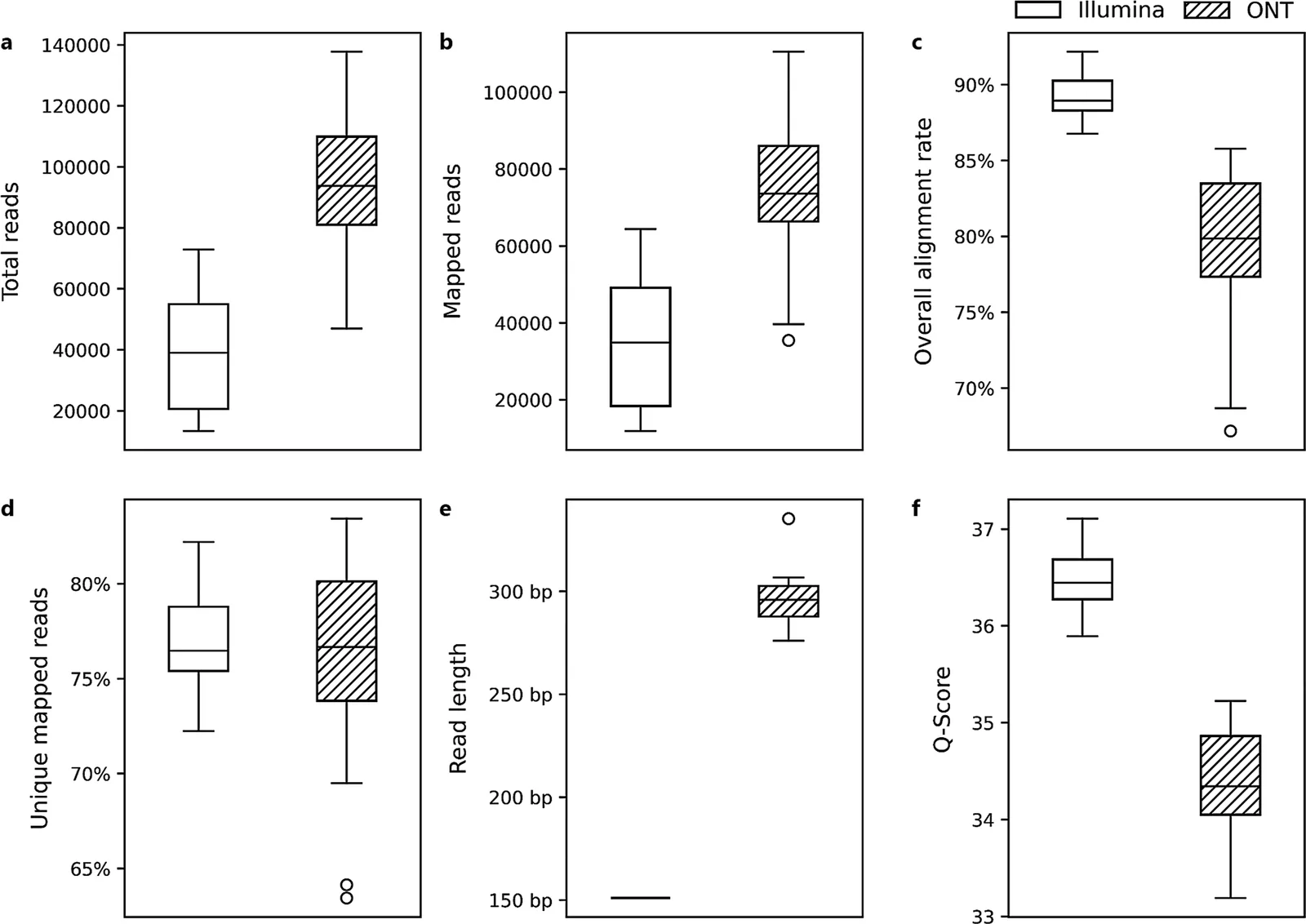
Comparison of metrics between Illumina (white) and Oxford Nanopore (ONT, hatched) sequencing. **a** Total reads. **b** Mapped reads. **c** Percentage of mapped reads. **d** Percentage of uniquely mapped reads. **e** Average read length. **f** Q-score. Boxplots indicate median, interquartile range, and outliers.

### Allelic Diversity of the MH Panel

In total, 358 different alleles at the 54 MH loci were identified in the study population of 960 blood donors. The numbers varied from one MH (MH01CP007) with three alleles, 9 MHs with four alleles, 14 MHs with five alleles, 9 MHs with six alleles, 10 MHs with seven alleles, 2 MHs with eight alleles, and 5 MHs with nine alleles (shown in Fig. 3 and online suppl. Table 4). In addition, four MHs (13qA, MH07CP004, MH-27, MH-31) showed very high numbers of alleles (>10). Overall, the majority of 42 MHs showed between four and seven different alleles, indicating a reasonable allelic diversity. The overrepresentation (>60%) of a single allele was observed for nine MH markers, whereas the other markers exhibited a more balanced distribution of multiple alleles within the population. Pronounced differences are evident in both the number and the relative contribution of rare alleles. The allele frequency was low (0.01–0.001) for 81 of the 358 (22.6%) alleles, and 36 MH loci revealed at least one low-frequency allele. A very low allele frequency (<0.001) was observed for 42 alleles (11.7%) at 28 MH loci. These 123 rare alleles represented a substantial proportion (34.4%) of the total allelic variation.

**Fig. 3.**
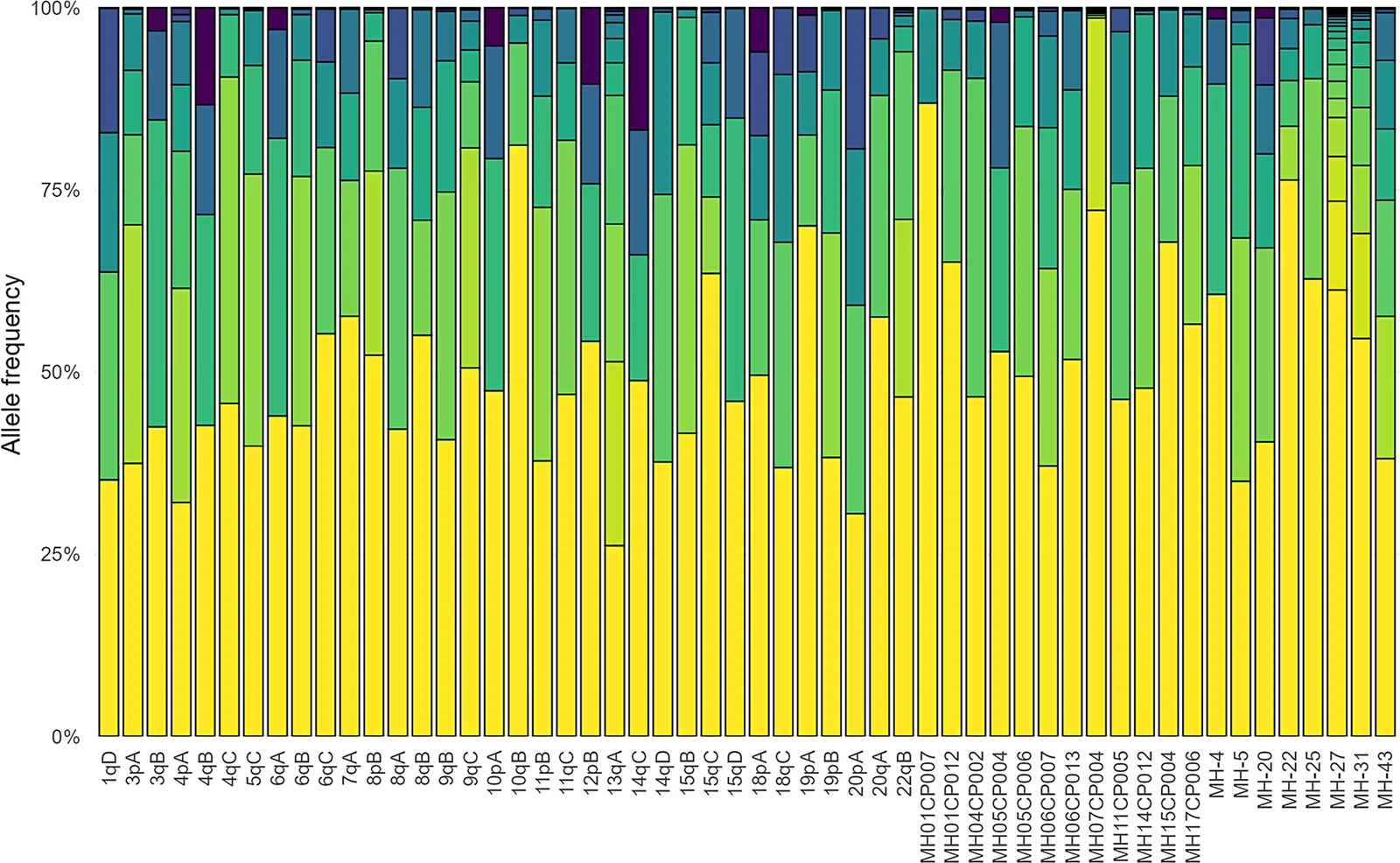
Allele frequency distribution of the examined MHs. For each marker, the relative frequencies of the observed haplotypes (alleles) are shown as stacked bars. Colors indicate the relative allele frequency (yellow for the most frequent and dark blue for the less frequent). The sum of allele frequencies per marker equals 100%.

Important statistic parameters including PIC, PD, HWE, H_e_, and A_e_ were calculated for evaluation of the MH panel (shown in Fig. 4 and values listed in online suppl. Table 5). The PD values were >0.6 for 52 loci (range 0.64–0.96; mean 0.87 SD ± 0.08), showing only minor locus-specific variation. MH01CP007 and 10qB revealed lower values of 0.40 and 0.54, respectively. The *p* values for the HWE indicated significant deviation from equilibrium for 15 loci (*p* < 0.05), whereas 39 loci showed no significant deviation (range 0.050–0.994). H_e_ and PIC values were generally moderate to high for the marker panel and displayed substantial inter-locus variability. A high PIC of >0.6 (0.61–0.77; mean 0.66 ± SD 0.04) was found for 23 markers, whereas 29 markers revealed a moderate PIC between 0.34 and 0.60 (mean 0.52 ± SD 0.07). The markers 10qB and MH01CP007 had low PICs of 0.29 and 0.20, respectively. The H_e_ was high (>0.6) for 41 MHs (0.601–0.797, mean 0.667 ± SD 0.043) and moderate for 12 MH (0.320–0.585, mean 0.497 ± SD 0.079). Only MH01CP007 showed a lower H_e_ of 0.228. For 33 MH loci, the effective number of alleles was moderate with A_e_ values <3.0 (range 1.295–2.951, mean 2.470 ± SD 0.559) (shown in Fig. 5). A higher diversity was observed for 21 MHs with A_e_ values between 3.02 and 4.93 (mean 3.56 ± SD 0.53). The average A_e_ value over all markers was 2.87 ± SD 0.83, reflecting the predominance of low to moderate allele numbers in the dataset. Overall, the majority of MHs showed high levels of allelic diversity and discriminatory power.

**Fig. 4.**
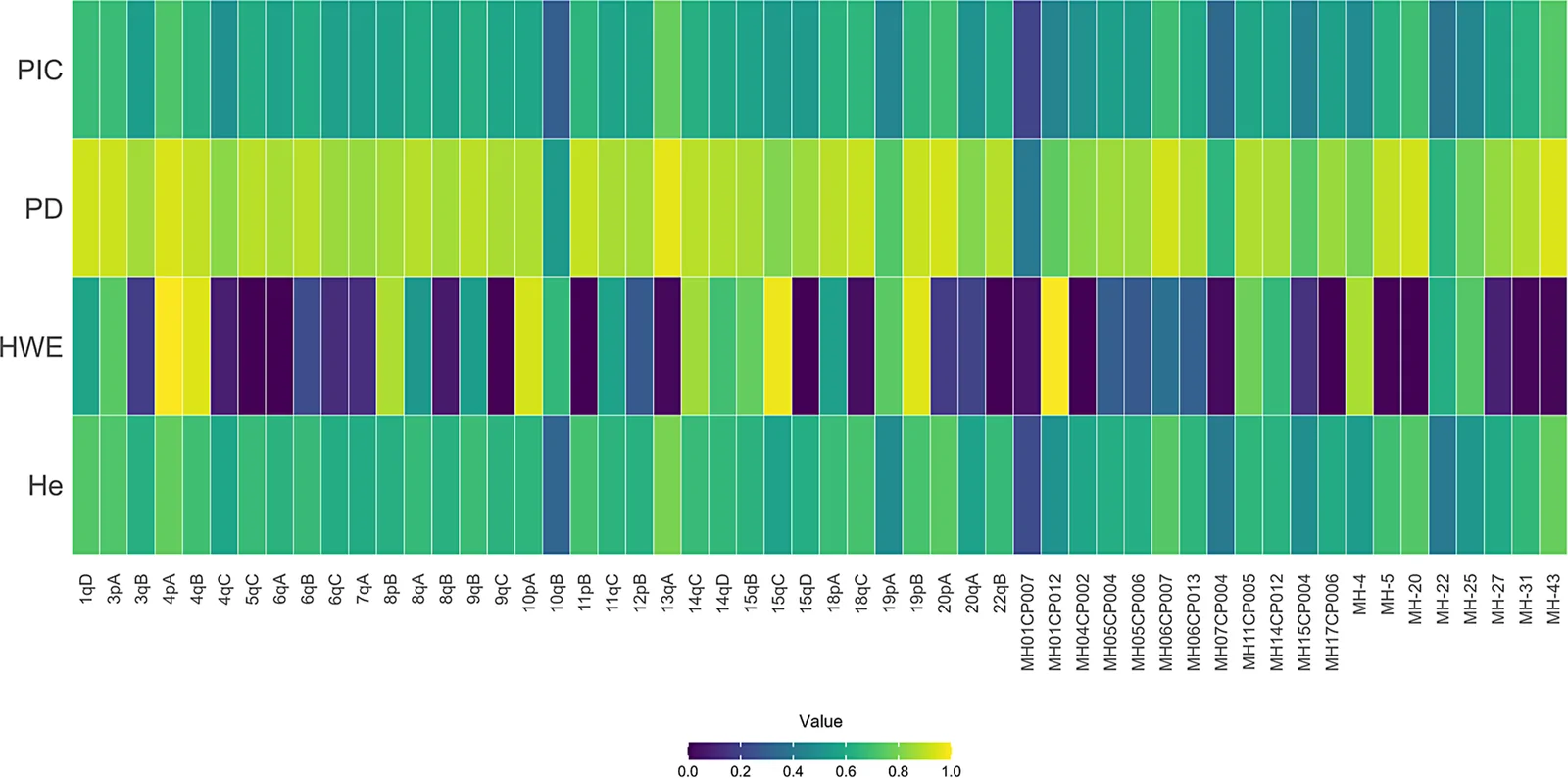
Heatmap of statistic parameter for the analyzed MHs. Polymorphism information content (PIC), power of discrimination (PD), Hardy-Weinberg equilibrium (HWE, *p* value) and expected heterozygosity (He) are shown for each marker. Color intensity represents normalized values ranging from 0.0 (dark blue) to 1.0 (yellow).

**Fig. 5.**
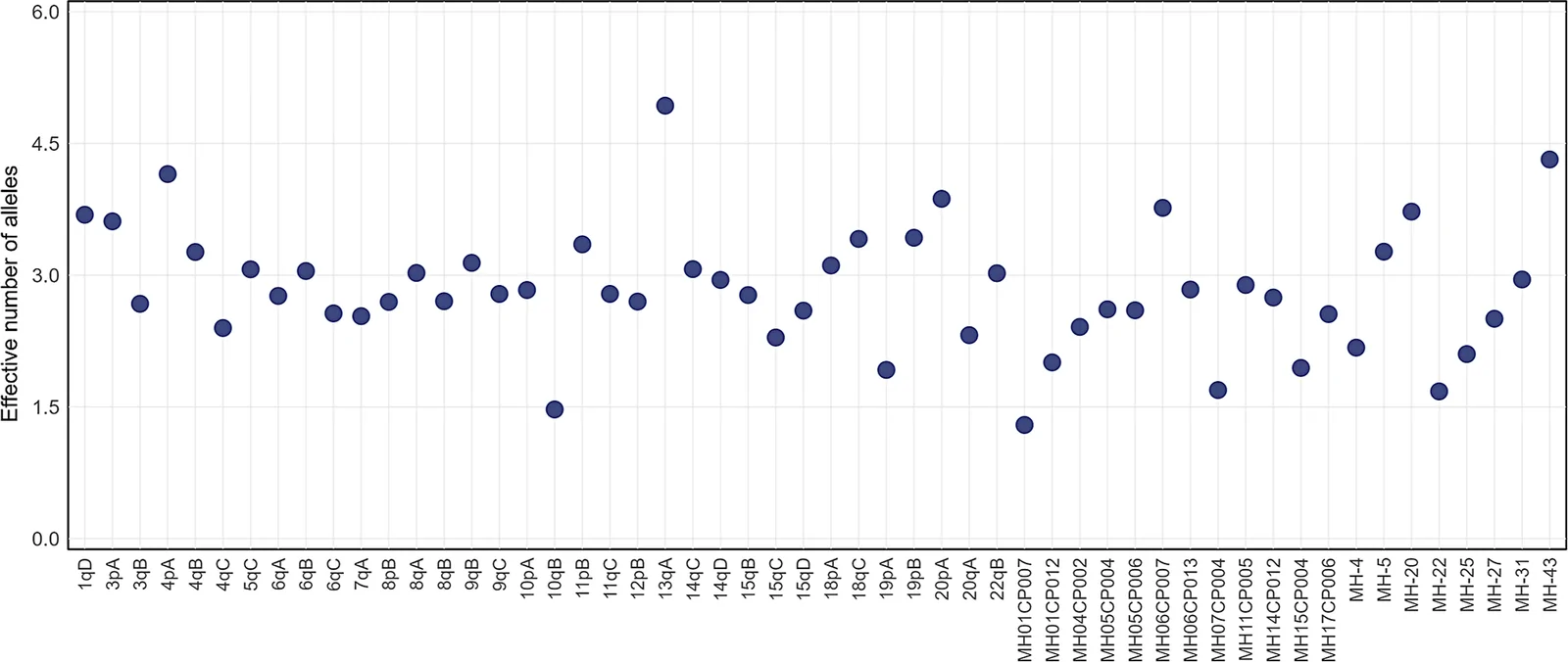
The effective number of alleles (A_e_) calculated for the 54 MHs.

### Additional SNVs

In the retrospective analysis of 960 sequenced samples, additional SNVs were identified in 23 MH loci that were not part of the originally described MH definition (Table 2). These additional variants were consistently observed in numerous samples. Most of the additional SNVs were located in close proximity but outside the defined MH region. As an example, the 4qB marker was originally defined by three SNVs (rs60652198, rs6532796, rs3828541). An additional SNV (rs6532795) was identified in close proximity to the MH region (shown in Fig. 6). At seven MH loci (8 pB, 8qA, MH06CP007, MH06CP013, MH07CP004, MH-31, MH-43) the additional SNVs were found in between the outer SNVs (Table 2). Thus, the additional SNVs expand the variability of the markers without having been included in the original MH descriptions. The results demonstrate the potential of extended sequencing approaches for a more detailed characterization of the MH markers.

**Table 2.** MH loci with additional SNVs within the sequenced amplicons

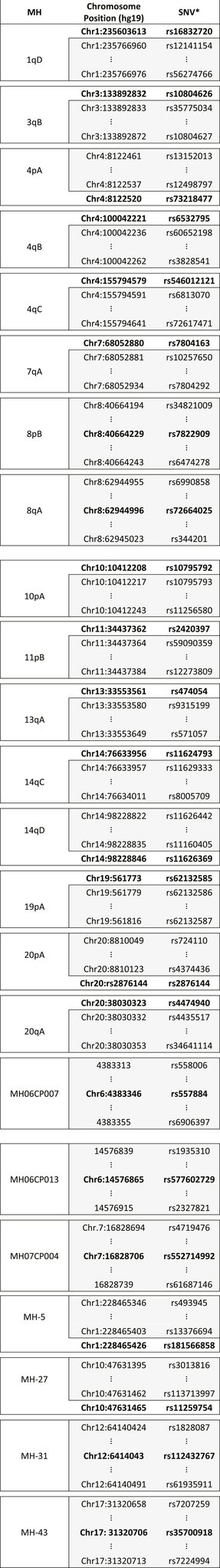

*Additional SNVs (bold numbers) were located outside or within a MH locus (gray box).

**Fig. 6.**
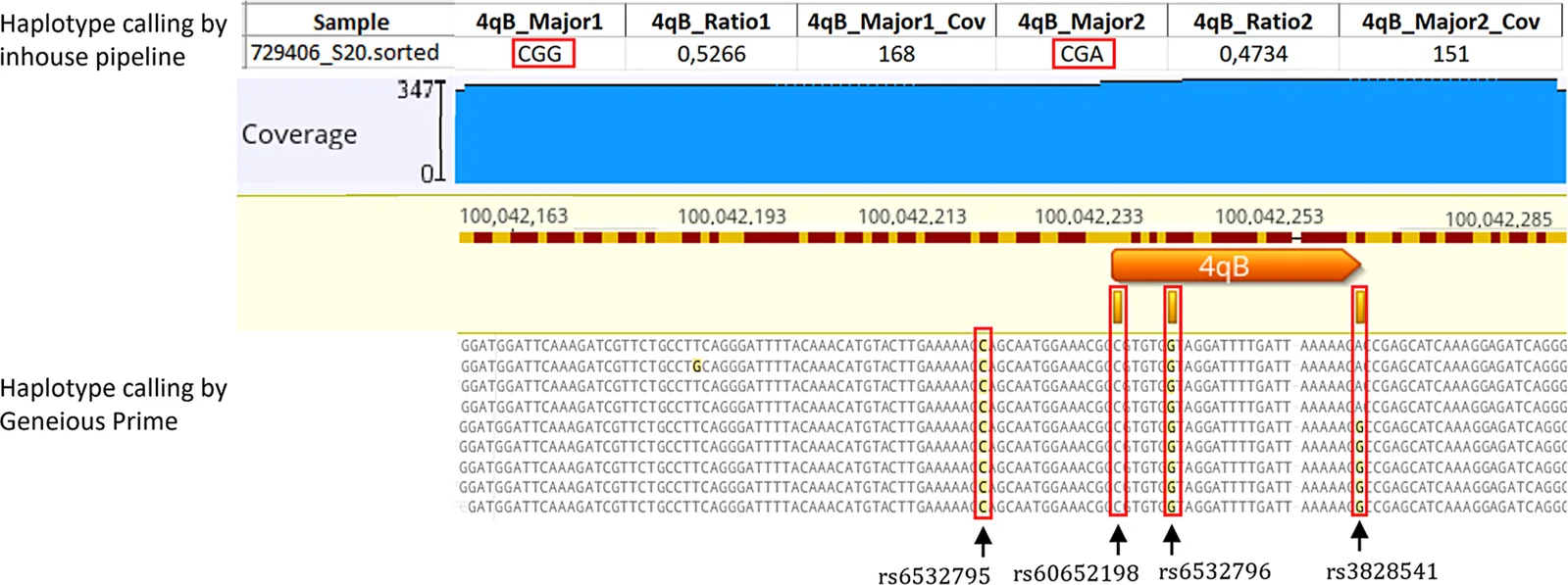
Representative example of haplotype calling for three SNVs (rs60652198, rs6532796, rs3828541) at the 4qB locus using an in-house pipeline (top) and Geneious Prime software (bottom). The identified major haplotypes (CGG and CGA), their relative proportions and read coverage are shown. The polymorphic positions of the 4qB region with regard to the reference genome (GRCh37, hg19) are highlighted. A further polymorphic SNV (rs6532795) was located in close proximity but outside the MH region and exhibits a reported allele frequency of 0.687 in the Caucasian population (1000 Genomes Project).

## Discussion

Our comprehensive characterization of genetic measures of a 54 MH marker panel was based on sequencing data from 960 individuals. Analysis of the allele frequencies revealed pronounced locus-specific heterogeneity, which is considered a characteristic feature of MHs. While some loci were dominated by single alleles, most loci exhibited effective allele numbers (A_e_) between 2.5 and 4, close to the nominal allele count, indicating a relatively even distribution of haplotypes. Loci with higher numbers of alleles (*n* > 10), however, displayed substantially lower A_e_ values, and the usable diversity was lower than suggested by the nominal allele count. MHs with a more balanced frequency of several alleles contributed significantly to the overall allelic diversity and better reflected population-specific differences than biallelic markers. The statistic parameters of the MH panel demonstrated high genetic diversity in the study population and were in close agreement with previous studies [21, 22]. Additional SNVs were identified in several MH loci. Some of these variants have previously been reported in international reference projects such as the 1000 Genomes Project; however, they have not yet been evaluated in the specific context of MH structures. Future investigations including additional populations are warranted as further SNVs are likely to be identified. The present findings highlight that a population-wide and MH-specific analysis of existing sequencing data has the potential to provide deeper insights into the genetic diversity of these markers.

A fully automated data analysis pipeline was established to ensure standardized, reproducible, and scalable processing of the large dataset. Following initial data preprocessing and quality control, raw data were converted into a uniform format and directly exported into an Excel-based analytical environment, which served as an interface between the automated data processing workflow and downstream statistical analysis. Within this environment, predefined calculation formulas were implemented to enable the automated determination of allele frequencies across all investigated loci and samples. Haplotype determination was subsequently performed based on the generated genotype data using defined filtering and quality criteria. The criteria were applied to identify validated allele combinations and to algorithmically infer the corresponding haplotype configurations. This approach ensured consistent and traceable calculations across the entire dataset, while excluding any manual intervention. Thus, the data analysis pipeline ensured high reproducibility and efficient processing of large sample numbers.

A previously established MH marker panel was successfully adapted to ONT sequencing, and the DNA profiling results were comparable with Illumina technology [23]. In our comparative analysis of 24 samples, the analysis of sequencing and mapping metrics revealed pronounced platform-dependent differences between Illumina and ONT data. Although ONT produced a higher absolute number of reads and mapped reads, Illumina achieved a significantly higher relative mapping efficiency, attributable to the higher base accuracy of the reads. The proportion of uniquely mapped reads was comparable between the two technologies, indicating that ONT reads attain sufficient genomic specificity despite their higher error rates. The similar total number of bases in the dataset suggested a comparable information density, while the markedly different read lengths reflected the intrinsic characteristics of the two sequencing platforms. In summary, both technologies can be used for generating reliable sequence data of MH panels. This has been shown also for the deconvolution of DNA mixtures [24].

A key objective of MH panel sequencing is to enable sensitive detection and specific evaluation of individual genetic profiles in mixed samples. In the context of allogenic hematopoietic stem cell transplantation, the MHs could enable chimerism detection by the identification and quantification of donor and recipient components. Recently, *Qama* et al. [25] demonstrated the use of a 24 INDEL marker panel analyzed by NGS for chimerism analysis following allogeneic stem cell transplantation. The recipient DNA was detectable at a low level of 0.1%. This is particularly relevant in the early post-transplant phase, when low residual levels of recipient DNA could be detected. Very similar detection limits are expected for MH panels because the NGS technology is almost the same as for the INDEL marker panel. So far, no distinct threshold of recipient DNA level associated with disease relapse has been determined, presumably, due to the different methods used for chimerism analysis [26, 27]. The broader use of high sensitivity NGS-based methods will allow the definition of a clinically relevant threshold.

The MH analysis also offers prospects for monitoring after organ transplantation. The detection of donor-derived cfDNA (dd-cfDNA) in blood samples of recipients was demonstrated for monitoring transplant rejection [28, 29]. In kidney transplantation, the specific dd-cfDNA detection enabled the early detection of immune-mediated graft injury and organ rejection [30]. A large panel of 266 biallelic SNVs was validated for clinical use [31]. The low allelic diversity of the SNVs demanded the high number of markers to ensure the donor-recipient discrimination and specific detection of dd-cfDNA. Because of the high allelic diversity, the number of MH markers required for a reliable chimerism detection is significantly lower. The short fragment length of approximately 150–170 bp of cfDNA must be considered for the primer design. The MH loci in our marker panel were selected and optimized for amplification in short fragments and, presumably, enable the amplification from cfDNA. The analysis of MHs amplified from cfDNA has been shown before in the context of noninvasive prenatal testing [32].

When using MH markers for diagnostic purposes, further limitations of the MH markers need to be addressed. Differences in allele and haplotype frequency, population-specific effects, and variable amplification efficiencies can limit the informative value of individual markers. Although a large marker panel increases discrimination power, it can unnecessarily increase the analysis efforts, costs, and complexity. Thus, the reduction of the 54 MH markers to the most informative 20 to 25 loci is a reasonable step to further optimization of the MH panel. Selecting the top 20–25 markers with the highest Ae values reduces the random match probability to 3.1 × 10^−10^ to 1.5 × 10^−13^, corresponding to a confidence of >0.99999999969%. Such a reduced panel achieves a discrimination power comparable to conventional 17–24 short tandem repeat marker panels, while enabling a significantly more efficient and cost-effective analysis [33, 34]. The panel size significantly affects the cost efficiency, i.e., the lower the number of markers in the panel, the higher the number of samples that can be analyzed per run. Since flow cells largely determine run costs, reducing panel size while maintaining coverage significantly lowers cost per sample. The differences in efficiency of amplification and sequencing can lead to unbalanced coverage of individual markers, which is particularly relevant for minor fractions at very low concentration. This will be evaluated by the analysis of mixed DNA samples. The goal is to achieve reliable low detection limits for cfDNA and gDNA. The optimized MH panel will then be validated for clinical use in stem cell and organ transplantation.

## Conclusion

The 54 MH markers represented a balanced set of loci with high discrimination significantly suitable for human identification. We established the genetic measures in the study population as a prerequisite for the next steps toward clinical application. This will include the validation of cfDNA analysis, the determination of detection limits in mixed samples, and the further optimization of cost efficiency by marker reduction. Both Illumina and ONT sequencing proved applicable for MH analysis; however, further investigation is needed to evaluate the platforms in the clinical setting of post-transplant monitoring.

## Statement of Ethics

The donors of this study gave written informed consent to use anonymized blood samples for research and development purposes. The Ethics Committee II of the Medical Faculty Mannheim, Heidelberg University, approved the use of anonymized blood samples for research and development purposes (Approval No. 2016-615N-MA).

## Conflict of Interest Statement

Christian Lang, Julia Wentzell, Lars Lodder, and Birgit Omengo were employees of inno-train Diagnostik GmbH. Peter Bugert was a member of the journal’s Editorial Board at the time of submission. Gabi Rink has no conflicts of interest to declare.

## Funding Sources

The Federal Ministry for Economic Affairs and Climate Action (BMWK) provided funding to P.B. (KK5702201CS4) and B.O. (KK5539602CS4) within the framework of the Central Innovation Programme for SMEs (ZIM). This study was further funded by the German Red Cross Blood Service Baden-Württemberg-Hessen gGmbH and by inno-train Diagnostik GmbH.

## Author Contributions

C.L. performed the laboratory work, evaluated the data, and wrote the manuscript. G.R. and J.W. assisted in laboratory work and reviewed the manuscript. L.L. supported the bioinformatic analyses and reviewed the manuscript. B.O. and P.B. designed the study, evaluated the data, and wrote the manuscript.

## Data Availability

All data generated or analyzed during this study are included in this article and its supplementary material files. Further inquiries can be directed to the corresponding author.
